# CRS + HIPEC combined with IP + IV chemotherapy for gastric signet-ring cell carcinoma: Case report of long-term survival

**DOI:** 10.1097/MD.0000000000022647

**Published:** 2020-10-09

**Authors:** Guo-Jun Yan, Zhong-He Ji, Gang Liu, Yan Li

**Affiliations:** Department of Peritoneal Cancer Surgery, Beijing Shijitan Hospital, Capital Medical University, Beijing, P. R. China.

**Keywords:** cytoreductive surgery, gastric cancer, hyperthermic intraperitoneal chemotherapy, intraperitoneal chemotherapy, peritoneal surface malignancy, signet-ring cell carcinoma

## Abstract

**Rationale:**

Signet ring cell carcinoma of the stomach is prone to relapse and metastasis after traditional surgical treatment, and the prognosis is also poor. We improved the concept of treatment and conducted cytoreductive surgery (CRS) plus hyperthermic intraperitoneal chemotherapy (HIPEC) combined with intraperitoneal (IP) and intravenous (IV) chemotherapy for a gastric signet-cell carcinoma patient.

**Patient concerns:**

A 65-year-old male patient with complaint of intermittent hematemesis for over 10 days was referred to our hospital for treatment. The patient developed hematemesis of 800 mL without obvious causes on May 27, 2015, accompanied by dizziness and amaurosis fugax. After the bleeding was stopped with medicinal treatment, diagnostic gastroscopy revealed an ulcer at the less curvature of the stomach, with biopsy pathology diagnosis as severe atypical hyperplasia, which was confirmed to be poorly differentiated adenocarcinoma by a second biopsy. In past medical history, the patient had 5 coronary stents implanted because of coronary atherosclerotic heart disease 3 years ago.

**Diagnosis:**

Gastric cancer (cT4NxMx) according to the patient's history and biopsy pathology.

**Interventions:**

the patient was treated surgery-based multidisciplinary treatments integrating CRS + HIPEC and IP + IV adjuvant chemotherapy. The CRS was curative distal gastrectomy with D2 lymphadenectomy, and HIPEC was cisplatin 120 mg plus mitomycin C 30 mg at 43 °C, for 60 minutes. Final pathological diagnosis of after surgery was: poorly differentiate adenocarcinoma with signet-ring cells, with invasion beyond the serosal layer and into the duodenum, 10/23 lymph nodes positive, nerve invasion, vascular tumor thrombi, Borrmann type IV, Lauren type diffuse. TNM stage was pT4aN3M0, IIIC. After operation, the patient received 6 courses of IV chemotherapy with oxaliplatin and 5-fluorouracil/Tegafur Gimeracil Oteracil Potassium capsules, and IP chemotherapy with docetaxel and carboplatin.

**Outcomes:**

Regular follow-up till July 20, 2020, revealed that the patient has a disease-free survival of over 61+ months.

**Lessons:**

CRS + HIPEC combined with IP + IV chemotherapy achieved long-term disease-free survival for this patient with gastric signet-ring cell carcinoma and deserve further study. This new treatment modality deserves appropriate consideration in routine clinical practice for patients with advanced gastric cancer.

## 1 Introduction

Although the incidence and mortality of gastric cancer (GC) are declining worldwide, East Asian remains highest in both incidence and mortality, where China, Japan, and South Korea account for 50% to 60% of the global GC burden.^[1]^ According to the statistics of National Healthy Commission of the People's Republic of China, in 2018, the mortality of GC reached 27.74/100,000 for males and 11.69/100,000 for females.^[2]^ Compared with Japan and South Korea, China lacks adequate nationwide early screening of GC. Patients are mostly in the advanced stage at presentation, and the proportion of early GC (around 15%) is much lower than that of Japan (68.9%) and South Korea (60.3%).^[3,4]^

Among all pathological types of GC, the signet-ring cell carcinoma in advanced GC has the highest malignancy and the worst prognosis. Moreover, the incidence of signet-ring cell carcinoma has been increasing.^[5]^ The treatment of gastric signet-ring cell carcinoma is still mainly based on surgical resection, combined with perioperative comprehensive treatment, but the effect is not satisfactory, with a median survival of 15.9 to 20.8 months.^[1,6]^ Simply extending surgery does not improve the prognosis of gastric signet-ring cell carcinoma.^[7]^ New treatment strategies should be explored.

Any development in new treatment strategies for signet-ring cell GC should be primarily based on tumor biology. The most prominent biological features of gastric signet-ring cell carcinoma are the strong invasive growth and the propensity towards abdominal-pelvic seeding, resulting peritoneal metastasis. Based on such understanding, systemic intravenous (IV) chemotherapy, intraperitoneal (IP) chemotherapy, either alone or in combination, have been explored.^[8,9]^ At the same time, greater efforts have been made to integrate more extensive surgery such as cytoreductive surgery (CRS) with more effective loco-regional chemotherapy such as hyperthermic intraperitoneal chemotherapy (HIPEC).^[10]^

In this report, we present our experience in CRS + HIPEC combined with IV + IP chemotherapy for a successful treatment of a patient with signet-ring cell GC, who is still a disease-free survival 61+ months after such integrated treatment.

## 2 Clinical presentation

A 67 years old man was referred to our hospital with a chief complain of “intermittent hematemesis for over 10 days”. The patient developed hematemesis of 800 mL on May 27, 2015, accompanied by dizziness and amaurosis fugax. After the bleeding was stopped with medicinal treatment, diagnostic gastroscopy revealed an ulcer at the less curvature of the stomach, with biopsy pathology diagnosis as severe atypical hyperplasia. In past medical history, the patient had 5 coronary stents implanted because of coronary atherosclerotic heart disease 3 years before. At physical examination, the body temperature was 36.0 °C, heart rates 72/min, blood pressure 125/85 mm Hg. The patient had pale conjunctivas and lips with moderate anemia manifestation. There were not positive signs in abdomen examination. Supraclavicular lymph nodes were not enlarged. No pelvic mass was detected on the digital rectal examination.

## 3 Laboratory workups

Tumor markers showed within normal range that alpha fetoprotein 3.75 ng/mL, carcinoembryonic antigen 3.6 ng/mL, carbohydrate antigen 6.75 U/mL, and carbohydrate antigen 6.5 U/mL. Routine blood test showed anemia with hemoglobin 81 g/L.

**Figure 1:**
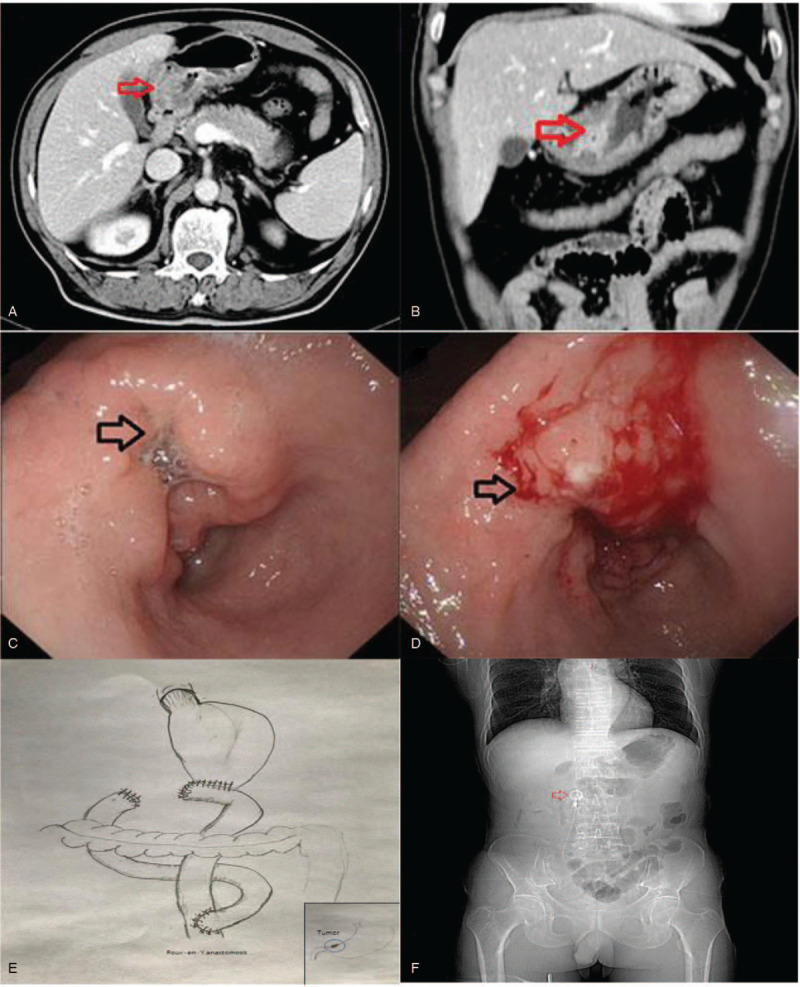
Preoperative abdomenal enhanced computed tomography (CT) test and endocopy. A: cross-section CT examination showed the gastric antrum tumor invaded the surrounding structures; B: coronary CT examination revealed a large mass in the gastric antrum in the stomach and obstruction; C: gastroscopy showing a large irregular ulcer in the antrum; D: ulcerative bleeding after gastroscopy. E: Roux-en-Y anatomosis after gastrecromy. F: X-ray of abdmen shows peritoneal pump.

Abdominopelvic computed tomography showed a mass on the gastric antrum, 3.6 cm × 2.4 cm, with uneven thickening of the gastric wall and the blurred surrounding adipose (Figs. 1A, 1B). No enlarged lymph nodes were found around the lesion.

A second gastroscopy showed an irregular ulcer about 4.0 cm × 5.0 cm at the gastric antrum (Fig. 1C, D). The lesion surface was uneven, the boundary was clear but irregular, and the tissue was brittle and easily bleeding. Biopsy pathology revealed signet ring cell carcinoma in the background of chronic gastritis with moderate intestinal metaplasia.

## 4 Diagnosis

The clinical diagnoses were:

(1) GC, at the antrum, signet-ring cell carcinoma, stage cT4NxM0, Borrmann IV;
(2) anemia, moderate degree;
(3) coronary atherosclerotic heart disease.

## 5 CRS + HIPEC based integrated treatment

A multi-disciplinary consultation was held. As the diagnosis was signet-ring cell GC (cT4NxM0) without distant metastasis, there was a high risk for peritoneal metastasis, because the serosa invasion was demonstrated on computed tomography. Moreover, potential peritoneal metastasis could have been developed based on the tumor biology and imaging studies. Therefore, CRS + HIPEC was suggested as a intent-to-cure treatment, to both reduce the risk of peritoneal metastasis and improve the possibility of radical resection.

On June 19, 2015, CRS + HIPEC was performed for this patient. Abdominal exploration found a hard tumor about 4 cm in diameter at the stomach angle with direct invasion into the lesser omentum. Multiple enlarged lymph nodes were palpated around the lesion. The CRS was radical gastrectomy to remove the distal 3/4 of the stomach, 2-cm proximal duodenum, greater and lesser omentum, and D2 lymphadenectomy. After the specimen was removed, intraoperative HIPEC was performed, with cisplatin 120 mg and mitomycin 30 mg, each dissolved into 3 L of saline, at 43 °C for 60 minutes. After HIPEC the anastomosis was performed by Roux-en-Y behind transverse colon (Fig. 1E). A peritoneal chemotherapy pump was implanted (Fig. 1F). 2 drainage tubes were placed on left subdiaphragm and duodenal stump. The operation took 375 minutes. The intraoperative blood loss was 400 mL, and blood transfusion were red blood cell 4 U and plasma 600 mL. The patient received comprehensive support and was discharged 13 days post-operation.

**Figure 2:**
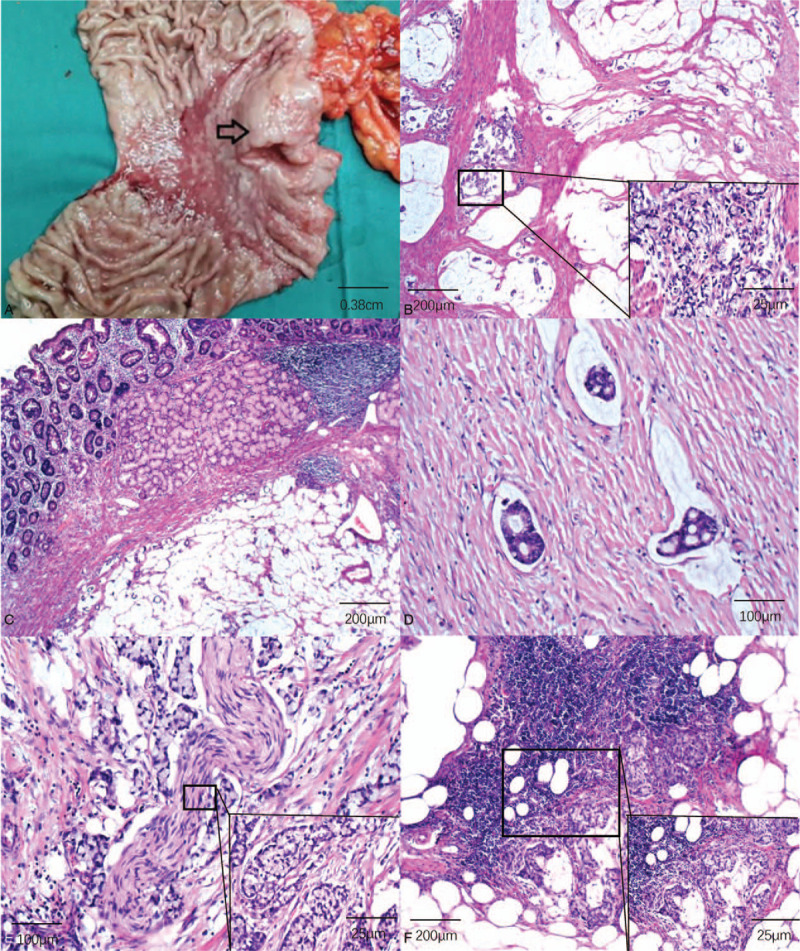
Postoperative pathology examination. A: gross patholgy presented large and deep ulcers with large mucosal erosions around it, showing precancerous lesions. B: signet-ring cells damaged the smooth muscle of the stomach wall (HE dyeing 50X, 400X); C: signet-ring cell inviaded pylorus and duodenum (HE dyeing 50X); D: angioma plugs in vessel (HE deying 200X); E: the tumor invadied nerve (HE dyeing 200X, 400X); F: lymphode metastasis, large amout of signet cells in the lympdode medulla (HE dyeing 200X, 400X).

Postoperative pathology showed a big irregular ulcerous mass about 4.0 cm × 4.0 cm in surface area and 1.5 in depth, with a wide zone of gastric mucosa erosion surrounding the lesion (Fig. 2A). The distal resection margin was about 1.0 cm and the proximal resection margin was about 5.0 cm from the lesion. Conventional histopathology showed gastric signet-ring cell carcinoma admixed with mucinous adenocarcinoma (Fig. 2B), invading the pylorus and the duodenum (Fig. 2C), the blood vessels to form tumor emboli (Fig. 2D), and the nerves (Fig. 2E). There were also cancerous nodules in the surrounding fibrous tissue, and among the 23 lymph nodes examined 10 were positive for metastasis (Fig. 2F). Immunohistochemical studies revealed that the tumor was positive for CK7, CK8, CK18, CK20, P53, MUC1, and MUC6, negative for HER2, PD1, and PD-L1, and the Ki-67 label index 80%. Therefore, the postoperative TNM stage was pT4aN3M0, IIIC. Lauren classification: diffuse type.

Postoperative treatments included IV + IP chemotherapies. The first cycle adjuvant chemotherapy was delivered on July 16, 2015, with IV chemotherapy: oxaliplatin 150 mg, calcium folate 300 mg and 5-fluorouracil 500 mg D1, and 5-fluorouracil 1000 mg D1-D2; and IP chemotherapy: paclitaxel 90 mg D1, and carboplatin 200 mg D1-D2. Blood test in August 3, 2015, showed that leukocyte 1.96 × 10^9^/L, neutrophil 0.8 × 10^9^/L, HB 73 g/L, platelets 36 × 10^9^/L, which revealed III° Bone marrow suppression.^[11]^ The patient improved after giving drug to stimulate white blood cell and platelet. Second cycle chemotherapy (August 10, 2015): oxaliplatin 100 mg D1 IV, Tegafur Gimeracil Oteracil Potassium capsules 60 mg bid D1-14 PO. Third cycle chemotherapy ( September 10, 2015): paclitaxel 90 mg D1, carboplatin 200 mg D1-D2 IP; Tegafur Gimeracil Oteracil Potassium capsules 60 mg bid D1-14 PO. Fourth to sixth cycle chemotherapy (SOX, October 12, 2015–December 1, 2015): oxaliplatin 100 mg D1 IV, Tegafur Gimeracil Oteracil Potassium capsules 40 mg bid D1-14 PO. Regular follow-up review after adjuvant chemotherapy.

## 6 Postoperative follow up

**Figure 3:**
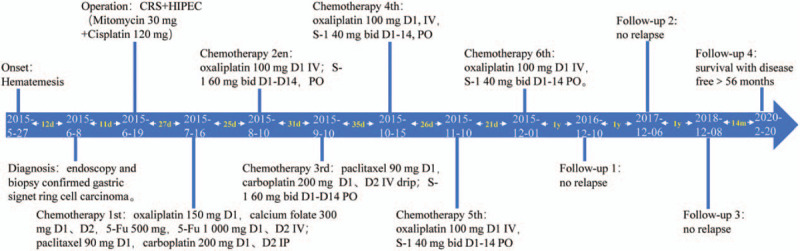
The procedure of treatment. The patient has survived over 56 months as to latest follow-up.

The patient has survived over 61+ months as to July 20, 2020 (Fig. 3). This case report was approved by the ethics committee of Beijing Shijitan Hospital, Capital Medical University and the informed consents were obtained from the patient.

## 7 Discussion

Gastric signet ring cell carcinoma accounts for 16% to 20% of GC incidence.^[6]^ It has been reported that the early signet ring cell carcinoma of the stomach has a better prognosis than the early non-signet ring cell carcinoma.^[12]^ Prognosis of advanced signet ring cell carcinoma is significantly worse than non-signet ring cell carcinoma.^[6,13]^ Signet ring cell carcinoma is an independent poor prognostic factor for GC. Signet ring cell carcinoma has the characteristics of lower age of onset, higher incidence in women, poor differentiation of cancer cells, prone to peritoneal metastasis, and occurrence in distal stomach compared with non-signet ring cell carcinoma.^[6]^ Once peritoneal metastasis occurs in GC, the difficulty of treatment increases, the prognosis is poor, and the natural survival time is less than 5 months. The median survival time after comprehensive multidisciplinary treatment is 15.6 months.^[14]^

Among newly diagnosed GC patients, 10% to 20% have peritoneal metastasis, and 50% to 60% of patients have recurrence of peritoneal metastasis after radical gastrectomy.^[15–17]^ Gastric signet ring cell carcinoma has a peritoneal metastasis rate of 20%. Signet ring cell carcinoma, lymph node metastasis, T stage T3-4, invasive GC, and younger than 60 years of age are all high-risk factors for peritoneal metastasis.^[17]^ According to Maehara et al^[16]^, hematogenous metastasis and peritoneal metastasis are the most common and the prognosis is the worst. National Comprehensive Cancer Network guidelines recommend chemotherapy for peritoneal metastasis from GC.^[18]^ The integrated treatment strategy of CRS + HIPEC is based on the hypothesis that CRS could remove the entire visible tumor and HIPEC could destroy potential tumor cells dropped during operation or micrometastasis. Some patients receiving such integrated treatment have even been cured.^[10]^ For patients with a high risk of recurrence, no targeted prevention strategies have been proposed. The integrated treatment strategy of CRS + HIPEC provides possibility for the prevention of GC peritoneal metastasis.

HIPEC has been applied to the treatment of GC with peritoneal metastasis, which has the following advantages:

(1) chemotherapy drugs directly act on primary tumors, metastases, and free tumor cells in the abdominal cavity;
(2) the blood-peritoneal barrier will slow down drug absorption and increase drug action time, especially macromolecular drugs; after entering the liver through the portal vein system and then entering the systemic circulation, the adverse effects of chemotherapy were reduced.

Hyperthermia has a synergistic effect with IP infusion chemotherapy, increasing the depth of drug penetration.^[19]^ Repeated IP chemotherapy can further improve the efficacy. A meta-analysis shows that perioperative IP chemotherapy for resectable GC can bring significant survival benefits, and that HIPEC during surgery is more effective than postoperative IP chemotherapy.^[20]^

This patient is an elderly man who has been evaluated for resectable GC but has a risk of peritoneal metastasis. He underwent radical gastrectomy plus D2 lymph node dissection plus HIPEC. Compared with postoperative HIPEC, HIPEC contributes to reduce the risk of IP implantation caused by surgical shedding, and can also kill potential invisible metastatic lesions in the abdominal cavity, achieving radical cure at the cellular and molecular level. Signet ring cell carcinoma, T stage T4a, with lymph node metastasis, nerve invasion and vascular tumor thrombi, Borrmann type IV, Lauren type diffuse, all are high risk factors for recurrence of peritoneal metastasis. Patients received IV plus peritoneal chemotherapy after 3 weeks of rest. The patient had III° myelosuppression after first cycle chemotherapy, which was considered to be related with higher doses of IV plus peritoneal chemotherapy. Therefore, we reduced the dose of chemotherapy and gave him 5 cycle IV + IP chemotherapy. There are no signs of tumor relapse at the most recent follow-up on March 20, 2020. It is CRS + HIPEC, which is the integrated treatment strategy that removes visible tumors at the histological level and kills potential residual tumor cells at the cellular level, as well as postoperative peritoneal combined with IV chemotherapy, that bring survival benefits for patient.

In summary, this paper reports the successful treatment of gastric signet ring cell carcinoma with CRS + HIPEC combined with IV + IP chemotherapy. In line with medical literature, this integrated treatment provides an option for treating gastric signet ring cell carcinoma and is worth further study.

## Author contributions

**Supervision:** Yan Li.

**Validation:** Yan Li.

**Visualization:** Zhonghe Ji, Gang Liu.

**Writing – review & editing:** Guojun Yan.

## Abbreviations

Abbreviations: AFP = alpha fetoprotein, CA125 = carbohydrate antigen 125, CA199 = carbohydrate antigen 199, CEA = carcinoembryonic antigen, CT = computed tomography, CRS = cytoreductive surgery, 5-Fu = 5-fluorouracil, GC = gastric cancer, HIPEC = hyperthermic intraperitoneal chemotherapy, IP = intraperitoneal, IV = intravenous, NCCN = National Comprehensive Cancer Network, RBC = red blood cell.
